# Perception and Action in Sports. On the Functionality of Foveal and Peripheral Vision

**DOI:** 10.3389/fspor.2019.00066

**Published:** 2020-01-09

**Authors:** André Klostermann, Christian Vater, Ralf Kredel, Ernst-Joachim Hossner

**Affiliations:** Institute of Sport Science, University of Bern, Bern, Switzerland

**Keywords:** gaze behavior, expert performance, gaze anchor, visual pivot, foveal spot, quiet eye

## Abstract

An optimal coupling between perception and action is crucial for successful performance in sports. In basketball, for example, a stable fixation onto the basket helps to gain precise visual information of the target to successfully throw a basketball into the basket. In basketball-defense situations, however, opposing players cutting to the basket can be detected by using peripheral vision as less precise information are sufficient to mark this player. Those examples elucidate that to solve a given task foveal and peripheral vision can be used to acquire the necessary information. Following this reasoning, the current state of our framework will be presented that allows one to predict the functionality of one or the other or both depending on the current situation and task demands. In more detail, for tasks that require high motor precision like in far-aiming tasks, empirical evidence suggests that stable foveal fixations facilitate inhibitory processes of alternative action parameterization over movement planning and control. However, more complex situations (i.e., with more than one relevant information source), require peripheral vision to process relevant information by positioning gaze at a functional location which might actually be in free space between the relevant information sources. Based on these elaborations, we will discuss complementarities, the role of visual attention as well as practical implications.

In sports, athletes have to solve various tasks that require different solutions. To make this clear, imagine, you would be standing on a basketball court at the free-throw line with the task to *shoot* the ball into the basket. After two shot attempts, however, it is likely that you must immediately return to your own basket to *defend* the opponents' offensive actions. Obviously, the first and second task demand different motor actions. But, do they also require different gaze behaviors? Ask yourself: When solving these two tasks, where would you look and attend at? In the shooting task, you might look at the basket, the rim, or maybe at your hands while performing the shooting action. And in the defensive situation, you could focus the ball carrier, one of your teammates or maybe the opponent you are responsible for. Are these behaviors substantially different and what might be underlying functions? Exactly these questions which touch upon eye-movement associated behavioral costs have been addressed by our research group (for on overview on the current state of the art, e.g., Williams and Jackson, 2019). In this Perspective Paper, we will summarize current (own) research, underlying mechanisms and theoretical frameworks of cost-reducing eye movements. Different to earlier publications (e.g., Vater et al., 2019b), a recently developed framework proposing the functionality of foveal *and* peripheral vision in sports will be presented which states the complementarity of these two behavior. Beforehand, a glimpse of the physiological basis of the human eye and gaze behavior will be provided.

## Structure and Movements of the Human Eye

Because of the retinal structure (i.e., distribution of photoreceptor cells), visual information can be received via foveal and peripheral vision. Foveal vision refers to the small area-rule of thumb: size of your thumbnail hold at sleeve length-at which visual information can be gathered with very high visual acuity. However, since the number of cones decreases with increasing eccentricity-i.e., the angular distance from the fovea-all visual information outside of this foveal area (up to 5° of visual angle) are being perceived as increasingly blurred (up to 90% at 40° eccentricity). Despite this low visual acuity in peripheral vision, the high amount of rod cells on this retinal area leads to a high motion sensitivity (Strasburger et al., 2011).

In order to process information with high visual acuity, humans use body-, head-, and eye-movements to reposition the fovea at specific regions of interest. The latter is further divided into saccades, smooth-pursuit eye movements, vergence, and vestibular eye movements. Humans mostly apply saccades which are rapid eye movements with velocities as high as 500° per s (Rayner, 1998). During saccades sensitivity to visual information is reduced, a phenomenon which is also known as saccadic suppression (Binda and Morrone, 2018). In contrast, during smooth-pursuit eye movements visual-information sensitivity is comparable to fixations-i.e., the period of time the eyes are relatively still-but they only occur when the eyes are following an object (Spering et al., 2011). Thus, the repositioning of the fovea is quite costly because of the information loss. To avoid these costs, it makes sense to assume, that, particularly in sports, which requires to act in a complex environment with severe timing and precision demands, eye movements become attuned to the tasks to be solved (a current overview on perceptual-cognitive skills in sports can be found, e.g., in Brams et al., 2019). We will now elaborate this line of thought by addressing the functionality of an optimal timing of eye movements as well the use of peripheral vision.

## The Use of Foveal Vision

When reviewing the literature about the optimal timing of saccades (e.g., dynamic saccade analyses) only little is known. For example, in their extensive meta-analysis, Gegenfurtner et al. (2011) reported <10 studies, which analyzed saccades. It was found that experts initiate their saccades later (i.e., they have shorter durations to fixate task-relevant areas) and show longer saccadic amplitudes. However, the actual timing of eye movements, e.g., in relation to motor actions, particularly is reflected in the gaze phenomenon called Quiet Eye (QE), which is defined as the final fixation of a task-relevant object in space *before* movement initiation (Vickers, 2007). High-skilled athletes have been found to deploy longer QE durations than low-skilled athletes (e.g., Vickers, 1996) and successful attempts have been associated with longer QE durations than unsuccessful attempts (e.g., Klostermann et al., 2013). Usually, these differences in QE duration result from earlier QE onsets (for an overview, Vickers, 2007).

The QE has been assumed to optimize cognitive processes like information processing (e.g., Williams et al., 2002), movement parameterization (e.g., Vickers, 1996), and attentional control (e.g., Vine et al., 2014) (for an overview on potential mechanism, Gonzalez et al., 2015). Recently, Klostermann et al. (2014) proposed an advancement of the existing models to account for the improbable fundamental assumption of increased cognitive demands in highly skilled athletes (see also “efficiency paradox,” Mann et al., 2016). Drawing on an inhibition mechanism as introduced by Neumann (1996) and Cisek and Kalaska (2010), it is suggested that the QE subserves a shielding mechanism that promotes the parameterization of the optimal task solution. Recent work has been supporting this mechanism, as for example in a study that revealed relations between QE duration and response-selection demands in a far-aiming task. The higher the number of optional targets the earlier the QE onset and the longer the QE duration (Klostermann, 2019).

Regarding high motor performance, the empirical evidence emphasizes the functionality of an optimized coupling between perception and action which allows to perceive sufficient visual information for inhibition processes in subsequent actions. However, gaze behavior in high-skilled athletes further suggests that not only visual information from the fovea (as for example the QE phenomenon) is of relevance. But, visual information from the periphery seems crucial as, for example, shown by Hausegger et al. (2019) for defensive behavior in martial arts.

## The Use of Peripheral Vision

Experimentally, we investigated possible functionalities of peripheral vision by making use of a multiple-object-tracking (MOT) task (Pylyshyn and Storm, 1988). In a series of studies (Vater et al., 2016, 2017a), an experimental paradigm was introduced, which allowed us to study peripheral-perception performance in dual task situations (i.e., a primary monitoring task and a secondary detection task). In detail, participants are required to track four targets among six distractors, which all move on linear paths in a virtual box that spans an area of 40° × 40° of visual angle. Crucially, participants track those four targets by means of a virtual centroid and detect target changes with peripheral vision (Vater et al., 2016, 2017a). The advantage of this paradigm is that it can be applied to sport-specific settings (e.g., the monitoring of multiple players) without constraining the natural gaze behavior (for the technological implementation, e.g., Kredel et al., 2015; Vater et al., 2017b).

Our findings showed that when required to track information from multiple locations, peripheral vision outperforms saccades, in particular, when simultaneously solving further tasks like motion-change detection (Vater et al., 2017a). However, peripheral-perception performance is limited by crowding, i.e., the close proximity between objects at (far) eccentricities, which requires increased spatial acuity and evokes re-positioning of the gaze and a shift of the virtual centroid in the respective direction. Moreover, in case of (unpredicted) event changes (like a collision between objects), saccades need to be initiated to visually confirm the anticipation of the outcome of this event (e.g., Vater et al., 2017c).

Consequently, the empirical evidence points at the functional role of peripheral vision in sports requiring the monitoring and detection of peripheral events, particularly in time-demanding and complex situations often found in combat and team sports. However, due to biological constraints-both foveal (eccentricity) and peripheral (acuity)-and task constraints optimized gaze behavior seems necessary to make use of foveal and peripheral vision in sports.

## Optimal Gaze Behavior in Sports

The hypothesis of an optimized gaze behavior was addressed by Vater et al. (2019b) who reviewed the use of peripheral vision in sports (for general eye-tracking applications in sports, e.g., Kredel et al., 2017). Based on the findings from 29 studies covering different types of sports and tasks, levels of expertise and methods applied, they suggested three different, task-dependent functionalities of peripheral vision as indicated by different gaze behaviors.

**Figure 1:**
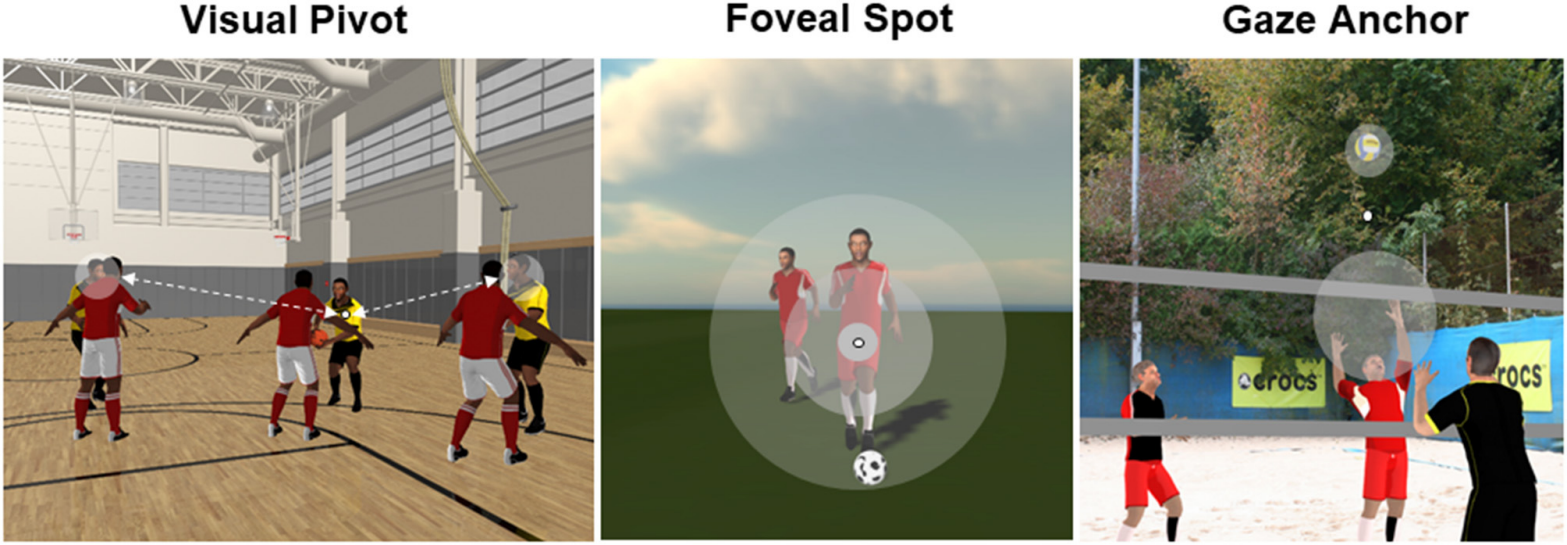
Three types of gaze behavior for the use of foveal and peripheral vision in sports (modified from Vater et al., 2019b). It should be noted that the small white spot illustrates the current gaze point and the grayish circled areas indicate potential attention locations as a function of respective relevant cues. Finally, in case of a foveal spot the breadth of attention can be modified as a function of the task demands.

If the task requires and the situation allows one to focus on one specific aspect-as for example the basketball free throw with high precision and low time demands-gaze is stabilized on one specific cue-like the rim of the basket-to get accurate visual information. If, however, the task still requires to process visual information with high spatial acuity but more than one cue prevails crucial information-like in 2 vs. 1 soccer situation when focusing the ball carrier-the attentional width can be increased if needed. This gaze behavior was labeled *foveal spot* (Figure 1, middle).

In a number of studies (e.g., Vansteenkiste et al., 2014), it was found that athletes locate their gaze in free space. For example, in beach-volleyball defense (Hossner et al., manuscript in preparation) instead of tracking the opponent and/or the ball, athletes locate their gaze at a position where the ball will be hit. It was assumed, that in these situations high spatial acuity is not required (otherwise gaze would be positioned to make use of foveal vision) and athletes use a *gaze anchor* (Figure 1, right) which allows one to monitor the movement of objects with peripheral vision (see also Vater et al., 2016). In contrast to the foveal spot, covert attention can be distributed to multiple objects. This, however, requires an optimal positioning of the gaze as it was shown by Hausegger et al. (2019) who found that the height of the gaze anchor on the opponent's body axis varies as a function of the attacking style in martial arts. Specifically, if attacks could be performed with arms and legs (Qwan Ki Do), athletes positioned the gaze anchor higher at the opponent's body compared to situations where mainly the legs are used to attack (Tae Kwan Do).

Finally, a *visual pivot* is used in situations that demand the processing of accurate visual information from a number of spatially distributed cues that cannot be covered by (para-)foveal vision (alone) and requires to reposition the fovea (Figure 1, left). As for the gaze anchor, it is hypothesized that the visual pivot is optimally located in-between the relevant information sources to allow for frequent fixation transitions by minimal saccadic costs. The visual pivot can be located in free space but also on information-rich cues, like the opponent's hip in soccer-defense situations (e.g., Vater et al., 2019a).

As emphasized by Vater et al. (2019b), crucially, one given situation does not evoke one of these gaze behavior only. As the situation evolves, different gaze behavior might interact, as for example in a basketball-offense situation in which the ball carrier, first might *anchor* his *gaze* centrally between his teammates to evaluate who might receive a pass. In the next moment, however, he might decide to shoot and to *foveally spot* the rim of the basket. Though, whether, indeed, the gaze positioned at the rim of the basket is used as foveal spot or as *pivot point* to still fixate the teammate which runs into an optimal playing position is difficult to determine by gaze data alone, because the gaze location might not indicate information processing.

## Complementarity and Future Research

As emphasized above, due to the complex interactions as they occur in sports with highly dynamic situational conditions and (motor) tasks with different demands, foveal and peripheral vision are necessary to be successful. Consequently, the mechanisms introduced are not mutually exclusive but, rather, complementary. We suggest that constraints inherent in the task require to balance an optimal positioning of the gaze with an optimally early onset of this fixation before movement initiation. The former depends on potential costs that occur when repositioning the fovea using a saccade (i.e., duration of information suppression) or when finding an optimal distance between adjacent objects to be monitored (i.e., perceptual impairment due to peripheral crowding) (see also Figure 1). The latter is necessary to facilitate (movement) parameterization via inhibition processes, thus, is highly dependent on the demands associated with the task. Consequently, an optimal gaze-anchoring location might not only be useful for processing peripheral information (i.e., perception) but also for the usage of this information for the following parameterization (i.e., motor control). Moreover, the positioning and the relative onset of this gaze-anchoring should be highly dependent on the associated cost-benefit equation. To the best of our knowledge, this hypothesis has not been addressed yet and will be pursued in future research projects in our laboratory.

Directly related to these questions, fundamental questions on the role of vision and attention in complex movement behavior should be made clear. As explained earlier, visual capabilities largely influence our gaze behavior and the use of foveal and peripheral vision. The interaction with attentional capabilities, however, and particularly research *directly* combining attention and vision measures in sport has been addressed only rudimentarily. Piras et al. (2017) suggested to study microsaccades as they might signal the direction of covert attention, thus, disentangling the current location of gaze from the location of attention (i.e., the microsaccade direction). But, this approach challenges eye-tracking technology, particularly when investigating complex movement behavior. Therefore, experimental approaches should be favored as proposed by Vater et al. (2019b) who suggested the systematic manipulation of peripheral information together with the prediction of gaze location. For example, based on previous research, it can be predicted that the ball carrier in soccer is the most likely gaze location. If one is able to react to a peripheral player's action without looking away from the ball carrier, this could be seen as an indicator of peripheral vision usage.

Finally, sport scientists should aim to transfer the obtained knowledge back into sports practice to improve the athletes' skills. The so-called perceptual training draws on the expert-performance approach (e.g., Ericsson and Smith, 1991) which requires to extract expert-like behavior, to empirically test its functionality, and to develop respective training programs which train these skills in less-skilled athletes (e.g., Vickers, 2007). Although a fair number of studies examined the effectiveness of these training programs, more often than not, research failed to show significant transfer effects (for an overview, e.g., Broadbent et al., 2019). In addition to issues with ecological validity (e.g., Hadlow et al., 2018), the training method is still debated. For example, research suggests that so-called gaze training by means of attentional cueing does not foster the learning of anticipation skills in beach-volleyball when compared to active control groups (e.g., Klostermann et al., 2015). Therefore, in the next years, increased efforts should be devoted to the theory-practice transfer answering questions like the trainability of expert-like gaze behavior in sports.

## Author Contributions

All authors listed have made a substantial, direct and intellectual contribution to the work, and approved it for publication.

### Conflict of Interest

The authors declare that the research was conducted in the absence of any commercial or financial relationships that could be construed as a potential conflict of interest.
